# Nature and Diffusion of COVID-19–related Oral Health Information on Chinese Social Media: Analysis of Tweets on Weibo

**DOI:** 10.2196/19981

**Published:** 2020-06-15

**Authors:** Zhuo-Ying Tao, Guang Chu, Colman McGrath, Fang Hua, Yiu Yan Leung, Wei-Fa Yang, Yu-Xiong Su

**Affiliations:** 1 Division of Oral and Maxillofacial Surgery Faculty of Dentistry The University of Hong Kong Hong Kong China (Hong Kong); 2 Key Laboratory of Shaanxi Province for Craniofacial Precision Medicine Research Research Center of Stomatology Xi’an Jiaotong University College of Stomatology Xi'an, Shaanxi Province China; 3 Applied Oral Sciences & Community Dental Care Faculty of Dentistry The University of Hong Kong Hong Kong China (Hong Kong); 4 Center for Evidence-Based Stomatology School & Hospital of Stomatology Wuhan University Wuhan, Hubei Province China

**Keywords:** COVID-19, dentistry, oral health, online health, social media, tweet, Weibo, China, health information

## Abstract

**Background:**

Social media has become increasingly important as a source of information for the public and is widely used for health-related information. The outbreak of the coronavirus disease (COVID-19) has exerted a negative impact on dental practices.

**Objective:**

The aim of this study is to analyze the nature and diffusion of COVID-19–related oral health information on the Chinese social media site Weibo.

**Methods:**

A total of 15,900 tweets related to oral health and dentistry information from Weibo during the COVID-19 outbreak in China (December 31, 2019, to March 16, 2020) were included in our study. Two researchers coded 1000 of the total tweets in advance, and two main thematic categories with eight subtypes were refined. The included tweets were analyzed over time and geographic region, and coded into eight thematic categories. Additionally, the time distributions of tweets containing information about dental services, needs of dental treatment, and home oral care during the COVID-19 epidemic were further analyzed.

**Results:**

People reacted rapidly to the emerging severe acute respiratory syndrome coronavirus 2 threat to dental services, and a large amount of COVID-19–related oral health information was tweeted on Weibo. The time and geographic distribution of tweets shared similarities with epidemiological data of the COVID-19 outbreak in China. Tweets containing home oral care and dental services content were the most frequently exchanged information (n=4803/15,900, 30.20% and n=4478, 28.16%, respectively). Significant differences of public attention were found between various types of bloggers in dental services–related tweets (*P*<.001), and the tweets from the government and media engaged the most public attention. The distributions of tweets containing information about dental services, needs of dental treatment, and home oral care information dynamically changed with time.

**Conclusions:**

Our study overviewed and analyzed social media data on the dental services and oral health information during the COVID-19 epidemic, thus, providing insights for government organizations, media, and dental professionals to better facilitate oral health communication and efficiently shape public concern through social media when routine dental services are unavailable during an unprecedented event. The study of the nature and distribution of social media can serve as a useful adjunct tool to help make public health policies.

## Introduction

The outbreak of the coronavirus disease (COVID-19) caused by severe acute respiratory syndrome coronavirus 2 (SARS-CoV-2), first identified from Wuhan, Hubei Province, China, has almost swept across the whole world and constituted a public health emergency of “pandemic” proportions [1]. SARS-CoV-2 is the third zoonotic human coronavirus emerging in this century after severe acute respiratory syndrome–related coronavirus and Middle East respiratory syndrome–related coronavirus (MERS-CoV) [2]. As a global pandemic, COVID-19 has affected people from more than 200 countries and regions, leading to 2,719,897 laboratory-confirmed cases and 187,705 deaths as of April 25, 2020 [3]. The total cases outside of China has outnumbered China more than 20 times. Strict prevention measures and effective therapeutics are urgently needed for the control of the pandemic.

COVID-19 has posed a particular threat to the practice of dentistry. With the identification of SARS-CoV-2 in the saliva of patients who are infected [4] and the transmission of COVID-19 through asymptomatic carriers [5-7] or potential patients in incubation status [8], awareness of the risk of COVID-19 spreading during dental procedures was of considerable concern among dental professionals [9-11]. Moreover, droplets and aerosols generated from high-speed dental handpieces, ultrasonic instruments, or 3-way syringes has the potential for direct or indirect cross transmissions of coronavirus between patients and dental care providers [12]. Therefore, during the early and outbreak stages of the COVID-19 epidemic in China, a majority of dental practices were suspended and most of the routine dental services were not available for the public, resulting in the inconvenience for potential patients to seek dental treatment. During this special period, social media played an important role in the exchange of dentistry- and oral health–related information among the public.

The internet, especially social media, is becoming increasingly important as a source of information for public health issues since it provides free and immediate access to large volumes of data [13]. In the past decade, social media has not only changed the pattern of spread for health-related information and the communication mode between patients and health care providers, but also drawn great attraction from researchers to study the distribution of diseases [14], the diffusion of health-related information and misinformation [15,16], the public reactions to health events [17-19], and more. Accumulating studies using social media for health care research have been published annually, providing insights for public health surveillance or helping develop health policy [14,20-23]. During the COVID-19 epidemic, the social media search index was identified as a promising predictor of new cases of COVID-19 infections [24].

Sina Weibo, similar to Twitter, is the most popular online microblog platform in China. Weibo allows its users to tweet or retweet messages optionally with links, pictures, or videos attached. The public reactions of Chinese people to the MERS-CoV and H7N9 outbreaks were significantly strong on Weibo [17]. Considering that the COVID-19 outbreak has exerted an impact on dental practices, we proposed the following research question: how was dentistry- or oral health–related information during the COVID-19 epidemic “tweeted” and communicated about on Weibo? The aim of this study was to investigate the nature and diffusion of COVID-19–related dentistry or oral health information on Weibo and determine the public reactions to tweets with this content, thus, providing an overview and reflection of the supply and demand of dental services under the epidemic on social media.

## Methods

### Study Design and Search Strategy

A study of “COVID-19-related oral health information” tweets on Sina Weibo was performed. A new anonymous Weibo account was created with only the name, gender, and date of birth provided upon registration. Using a new account without search histories, previous likes, or friends can avoid preferential links promoted by Weibo. Four keywords related to COVID-19 (pneumonia of unknown cause, coronavirus, COVID-19, and epidemic) and two keywords for dentistry (stomatology and dentistry) in Chinese characters were employed to search tweets about COVID-19 and dentistry or oral health on Weibo. Eight independent searches with a combination of one keyword for COVID-19 and the other for dentistry were carried out on March 17, 2020, through the new account. We selected December 31, 2019, as the start date of tweets since, on this day, the pneumonia of unknown cause (the name of COVID-19 at the time) in Wuhan was officially reported to the World Health Organization, and the first group of epidemiologists were dispatched by the Chinese Center for Disease Control and Prevention (CCDC) to support the control of this emerging infectious disease (EID) in Wuhan.

**Figure 1:**
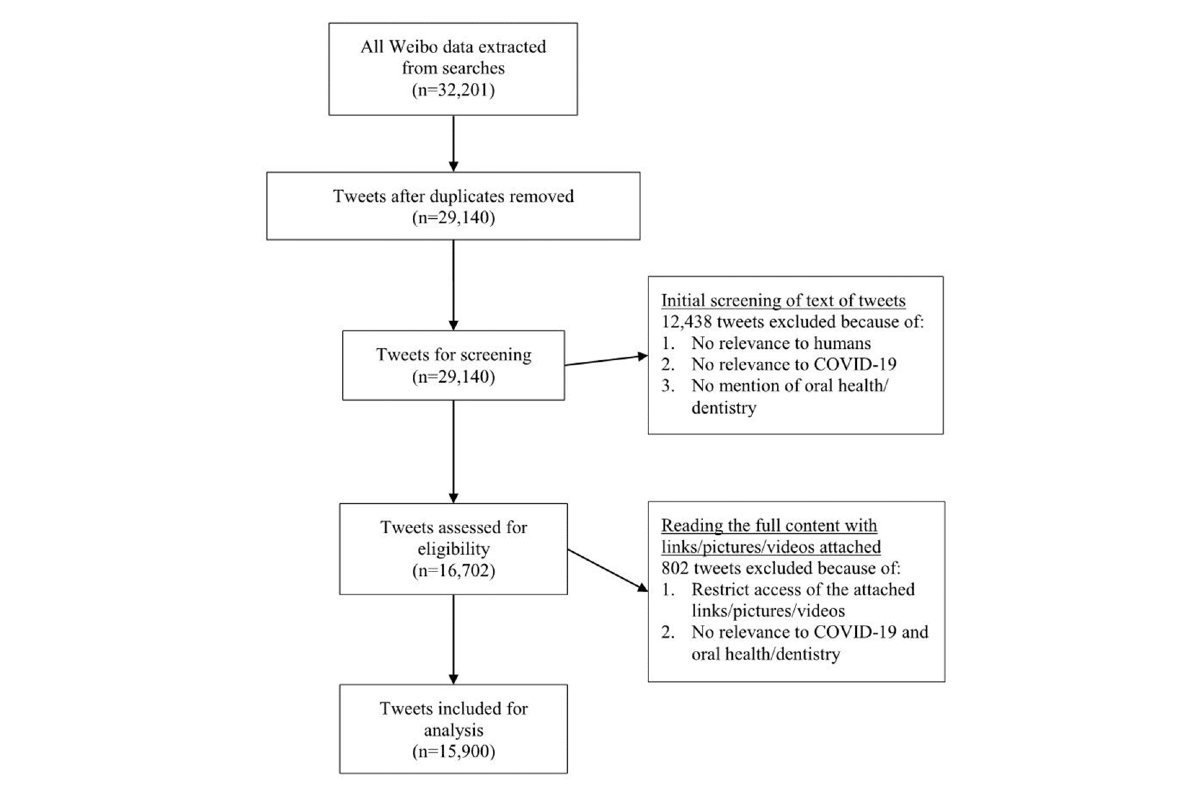
Flow chart of the study. COVID-19: coronavirus disease.

Figure 1 is the flow diagram used in our study. In total, 32,201 tweets in the first 77 days (December 31, 2019, to March 16, 2020) of the COVID-19 epidemic were identified. All Weibo data, including the full-text content; post time; numbers of likes, shares, and comments; and blogger information (name, ID, homepage website, number of followers, and personal introduction) of each tweet were extracted with a Python-based platform *Gooseeker*, which could retrieve all the tweets from each independent research through the account we created. A total of 29,140 tweets after removal of duplicates were scanned to exclude non–human-related and irrelevant results. After screening, there were 16,702 tweets included for further eligibility assessment, namely, reading the full content and the links, pictures, or videos attached to tweets as well. Restricted access of links, pictures, or videos were also excluded since only the information available for the public was assessed. Finally, the remaining 15,900 tweets were included for further analysis. The initial screening work and eligibility assessment was conducted by two researchers (ZT and GC) together.

### Coding Procedure

Two researchers (ZT and GC) with expertise in dentistry completed the coding. First, the two coders were asked to pilot the project by coding 1000 (6.28%) of the total 15,900 tweets to develop and refine the coding schemes for thematic categories. Two main thematic categories for all tweets were determined initially: COVID-19–related and oral health–related information. The information related to COVID-19 was further subtyped into five domains: epidemiology, pathology, symptoms, diagnosis, and prevention; the information related to oral health was subtyped into three domains: dental services, needs of dental treatment, and home oral care information. The tweets that were related to oral health and COVID-19 but inappropriate to be sorted into any of the categories were labelled “others.” The definition and examples of each category are shown in Multimedia Appendix 1.

Second, to test the feasibility and reliability of categories, a weighed Kappa test was used to assess interrater and intrarater agreement of coding between two researchers. Two researchers were asked to classify 200 randomly selected tweets and reclassify 2 weeks after the first coding. The results of the weighed Kappa test of the two researchers were 0.983 for interrater agreement and 0.994 (ZT) and 0.983 (GC) for intrarater agreement, which indicated excellent reliability of coding procedure.

Third, after agreement for the coding of the tweets was confirmed, all 15,900 tweets were randomly separated into two groups and classified by two researchers. The tweets were coded to more than one category if containing miscellaneous information. Tweets in each thematic category were divided into two types according to the numbers of followers (less than 1000 followers and 1000 or more followers).

### Postanalysis of the Included Tweets

The tweets of each day were counted and compared with COVID-19 daily new cases and deaths in China for time distribution analysis. Among 15,900 tweets, 1682 tweets with location information were analyzed for geographic distribution and compared with the regional distribution of total COVID-19 cases by March 16, 2020. The epidemiological data were obtained from the official website of the CCDC [25]. In addition, the tweets and retweets conveying similar information were extracted and grouped to analyze the topics of interest. The mean and SD of the numbers of likes, shares, and comments in each group were calculated.

Meanwhile, the tweets related to oral health information were further analyzed. Specifically, the time distribution of tweets containing information about the risks of COVID-19 transmission during dental procedures, notices of stopping all or part of dental services, need for dental treatment, home oral care, protective measures during dental services, and notices of restoring dental services during the COVID-19 epidemic were analyzed. The public needs for dental treatment and home oral care information tweeted on Weibo were further categorized and counted. The Kruskal-Wallis test was used to determine the differences of public reactions (numbers of likes, shares, and comments) to tweets from different types of bloggers (governments, media, dental clinics or hospitals, dentists or dental nurses, online health platforms, and others). In addition, the Mann-Whitney U test was used to compare the public reactions to tweets related to oral health information tweeted by the same types of bloggers.

All statistical analyses were carried out with Microsoft Excel (Microsoft Corporation) and SPSS software 18.0 (SPSS Inc), and *P*<.05 was considered significant.

## Results

### Time and Geographic Distribution of Weibo Tweets

**Figure 2:**
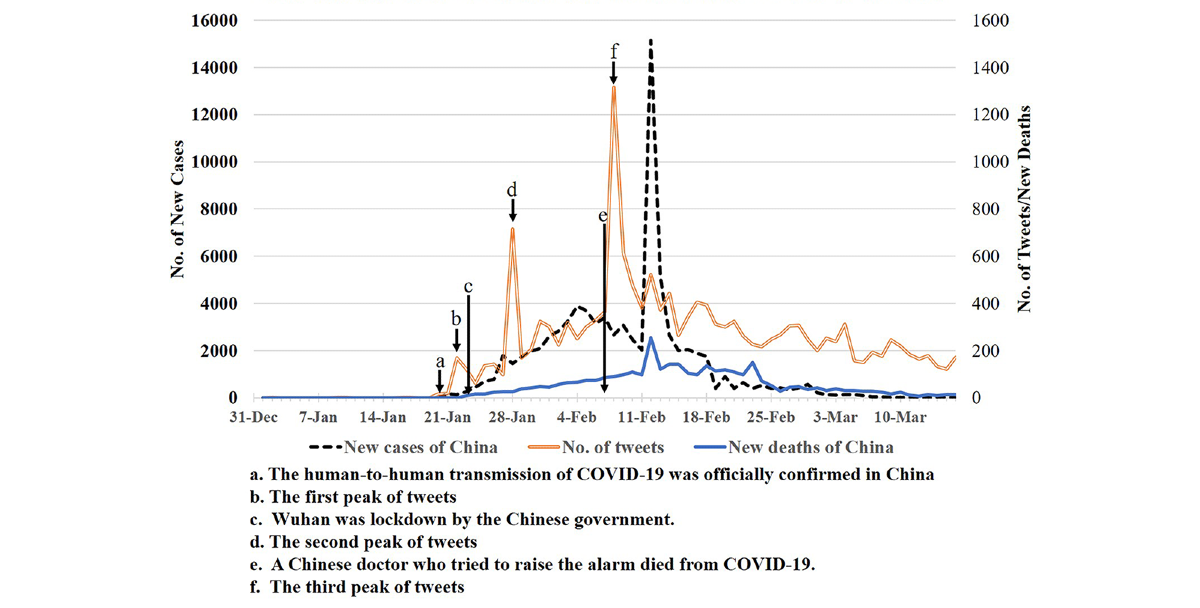
Time distribution of tweets and new cases and deaths of COVID-19 in China. COVID-19: coronavirus disease.

As shown in Figure 2, the time distribution of the Weibo data was similar to the distribution of new cases and deaths during this period of time. From December 31, 2019, to March 16, 2020, the daily count of tweets in Weibo was low during the first 20 days, then increased with fluctuations starting on January 20, 2020, presented three major peaks before mid-February, and finally gradually decreased and kept at a steady low level until March 16, 2020. The first peak on January 22, 2020, was between two milestone events of the COVID-19 outbreak in China, namely, official confirmation of human-to-human transmission of COVID-19 on January 20 and the start of the lockdown in Wuhan on January 23, 2020. The second peak on January 28, 2020, was composed of tweets and retweets refuting the misinformation for COVID-19 prevention. The highest peak of 1316 tweets on February 8, 2020, followed the death of Dr Wenliang Li, one of the first doctors who flagged the new coronavirus outbreak and raised alarms to the public. This peak occurred because retweets of the news that aerosol acts as a transmission route for COVID-19 as disseminated in a press conference of the Shanghai government office at 2 PM that day.

**Figure 3:**
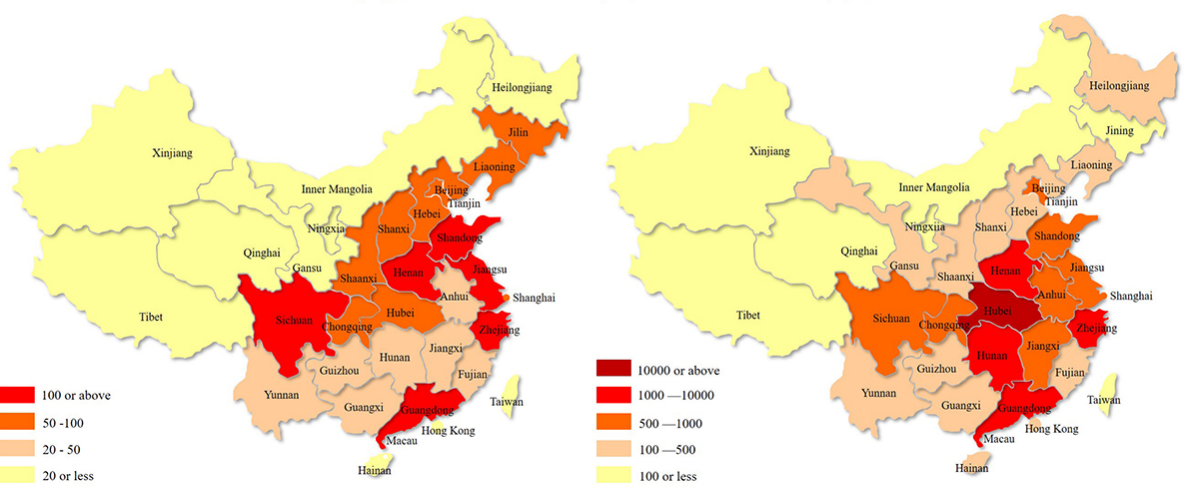
The geographic distributions of Weibo tweets (left) and total coronavirus disease cases (right) in the region.

Further analyses were conducted on 1682 tweets whose geographic distribution could be identified and compared to the regional distribution of total cases. The geographic location was optional for the users when posting tweets on Weibo. Therefore, only 1682 tweets included geographic location information. As demonstrated in Figure 3, the provinces (Zhejiang, Jiangsu, Guangdong, Shandong, Sichuan, and Henan) that witnessed the most tweets (>100) had more than 500 COVID-19 cases, higher than most of the other provinces in China. In northwest China, with no more than 100 confirmed cases in each province, tweets were less frequently distributed. More tweets were posted from central and eastern coastal provinces. However, tweets posted from Hubei, which was the original epicenter of the COVID-19 outbreak and had the most cumulative number of cases, surprisingly, were no more than 100.

### Thematic Distribution of Tweets and Highly Retweeted Information

**Figure 4:**
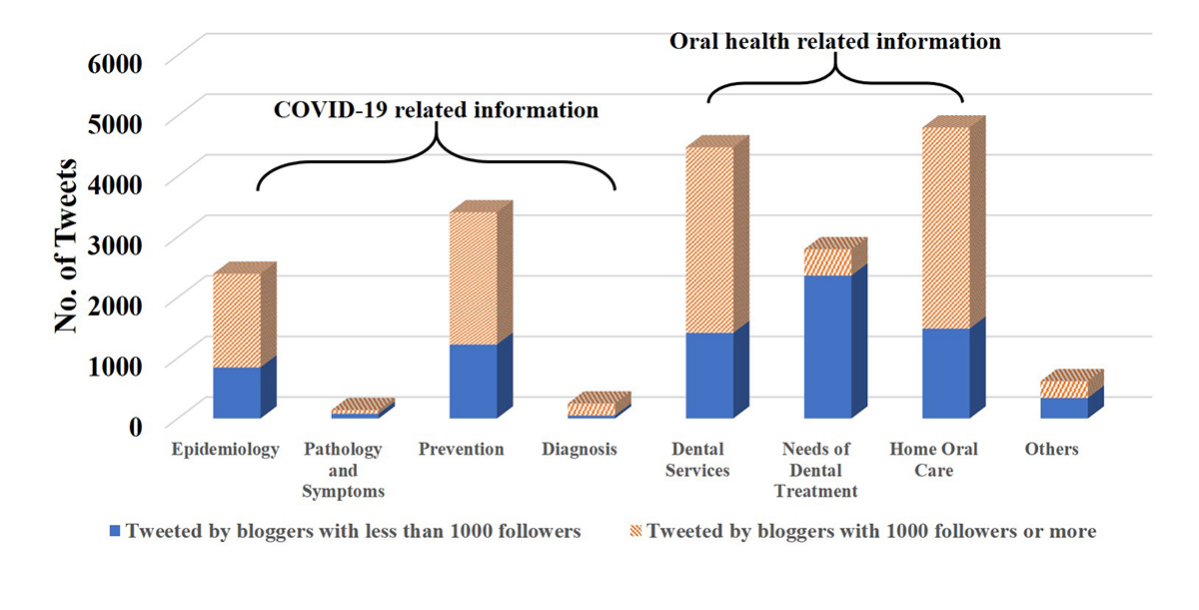
Thematic distributions of tweets with COVID-19–related oral health information. COVID-19: coronavirus disease.

Among 15,900 tweets included in our study, 79.81% (n=12,690) were oral health–related information and 38.86% (n=6180) contained background knowledge of COVID-19. As shown in Figure 4, the most commonly exchanged types of tweets related to oral health was home oral care information (n=4803, 30.20%), followed by dental service–related information (n=4478, 28.16%), the need for dental treatment (n=2793, 17.57%) during the epidemic, and other information about oral health or dentistry and COVID-19 (n=616, 3.87%). In terms of background knowledge about COVID-19, information about the prevention of COVID-19 (n=3404, 21.40%) witnessed the most tweets and retweets on Weibo, and epidemiology of COVID-19 (n=2390, 15.04%) was also common, while only a relatively small proportion of tweets mentioned aspects of pathology and symptoms (n=139, 0.87%) or diagnosis (n=247, 1.54%). The background information of COVID-19 (except the pathology and symptoms) was mostly tweeted by bloggers with more than 1000 followers. The dental services and home oral care information were highly tweeted by bloggers with 1000 or more followers, whereas the need for dental treatment were tweeted mostly by bloggers with followers less than 1000 (Figure 4).

**Figure 5:**
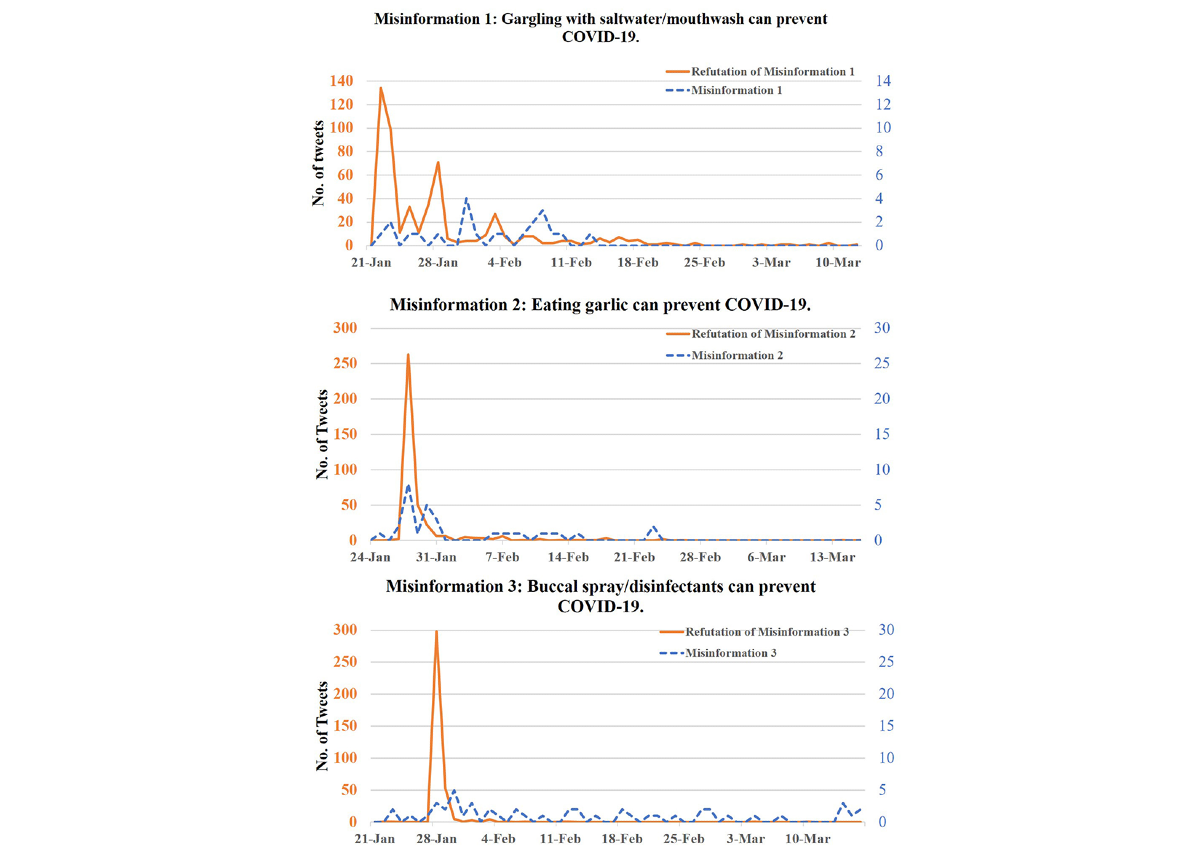
The time distributions of tweets related to misinformation for COVID-19 prevention and its refutations. COVID-19: coronavirus disease.

Some information was frequently tweeted or retweeted on Weibo and the top five pieces of widely diffused information were selected for evaluating the public reactions (Table 1). The most tweeted information was the news that aerosol acts as a transmission route of COVID-19, officially announced by the government office of Shanghai on February 8, 2020. There were 1406 tweets or retweets of this news or related aerosol information, and it gained 321.28 likes, 24.85 shares, and 19.70 comments on average. The second most tweeted information related to the risks of COVID-19 spread in dental clinics due to the aerosol generation, widely exchanged by dentists and dental clinics and hospitals, which averagely garnered 4.72 likes, 1.92 shares, and 1.31 comments. The next 3 groups of tweets were refutations of three pieces of widely spread misinformation of prevention measures for the coronavirus. As shown in Figure 5, the first misinformation that gargling with saltwater or mouthwash can prevent COVID-19 by lowering the level of coronavirus in the saliva spread among the public at an early stage of the epidemic (around the end of January). Soon the refutation of this misinformation was tweeted by the official platform “Weibo Refutes Rumours” and retweeted by others much more than the misinformation itself. A similar pattern was seen in another 2 groups of misinformation, namely, that eating garlic or applying oral spray or disinfectants can kill the coronavirus in oral cavities to protect from COVID-19 infection (Figure 5).

**Table 1:** Public reactions to highly tweeted information on Weibo.

Most highly tweeted information	Count, n	Public reactions		
		Likes, mean (SD)	Shares, mean (SD)	Comments, mean (SD)
The news propagandizing aerosol as a transmission route of COVID-19^a^	1406	321.28 (11,225.52)	24.85 (581.45)	19.70 (539.29)
Risks of COVID-19 spread by dental clinics due to the aerosol created by dental handpieces	659	4.72 (56.91)	1.92 (14.34)	1.31 (7.29)
Refutation of the misinformation that gargling with saltwater or mouthwash can prevent COVID-19	468	105.99 (1010.50)	27.27 (312.07)	9.98 (62.31)
Refutation of the misinformation that eating garlic can kill the novel coronavirus in the oral cavity	389	50.25 (639.83)	17.62 (237.45)	7.60 (84.71)
Refutation of the misinformation that oral spray/disinfectants can prevent COVID-19.	372	0.25 (1.88)	0.43 (2.74)	0.05 (0.32)

^a^COVID-19: coronavirus disease.

### Disruption of Dental Services During the Epidemic

**Figure 6:**
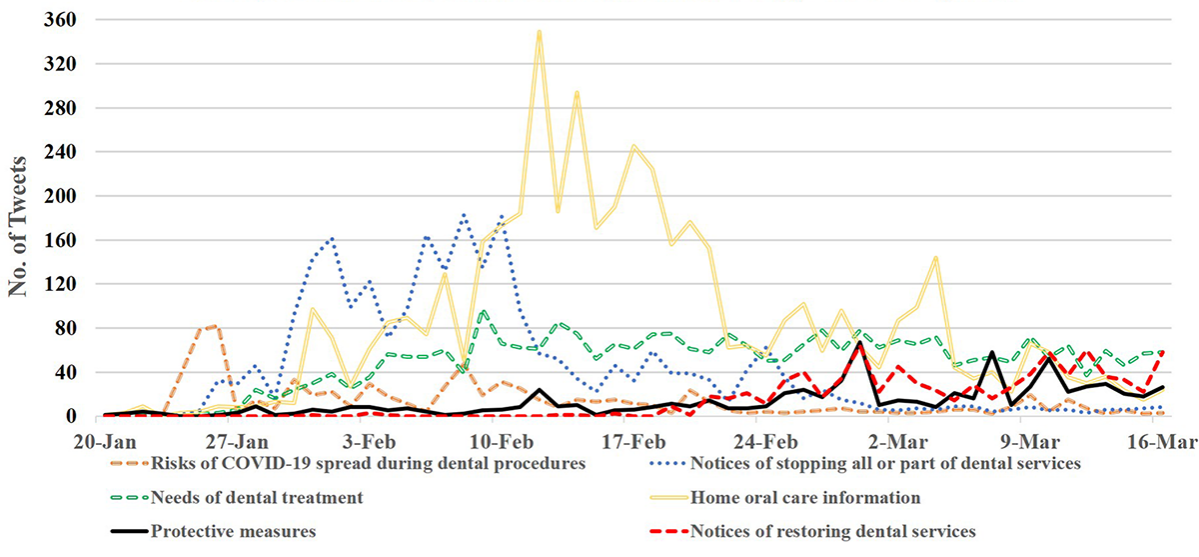
Time distributions of oral health–related tweets during the COVID-19 epidemic. COVID-19: coronavirus disease.

Tweets with different types of oral health–related information were distributed differently during the COVID-19 epidemic. As shown in Figure 6, the risk of COVID-19 spread during dental procedures was proposed during the early stage of the epidemic, and the associated tweets and retweets peaked on January 25 and 26, 2020, after the start of the Wuhan lockdown on January 23. During the first half of February, under the first level emergency response to COVID-19 in China, the government, public hospitals, and private dental clinics delivered notices to stop all or part of dental services to the public. Followed by this was the peak of information about home oral health care in the second half of February. From late February to early March, tweets associated with notices of restoring dental services and protective measures during dental treatment gradually increased due to the control of COVID-19 in China.

When the dental services were not available from the end of January to early March, many bloggers complained of oral problems and sought dental care on Weibo. There were a steady number of tweets (around 40-80 tweets/day; Figure 6) during this time period. As shown in Table 2 those complaining of toothaches or wisdom tooth problems (eg, pericoronitis, decay) were most common, followed by orthodontic-related problems, oral ulcers, and pediatric oral diseases; oral cancer and implants or prostheses only occupied a small proportion. Additionally, the rest of the tweets were related to seeking oral care for other dental diseases including tooth decay and gingival bleeding or without specific reasons.

**Figure 7:**
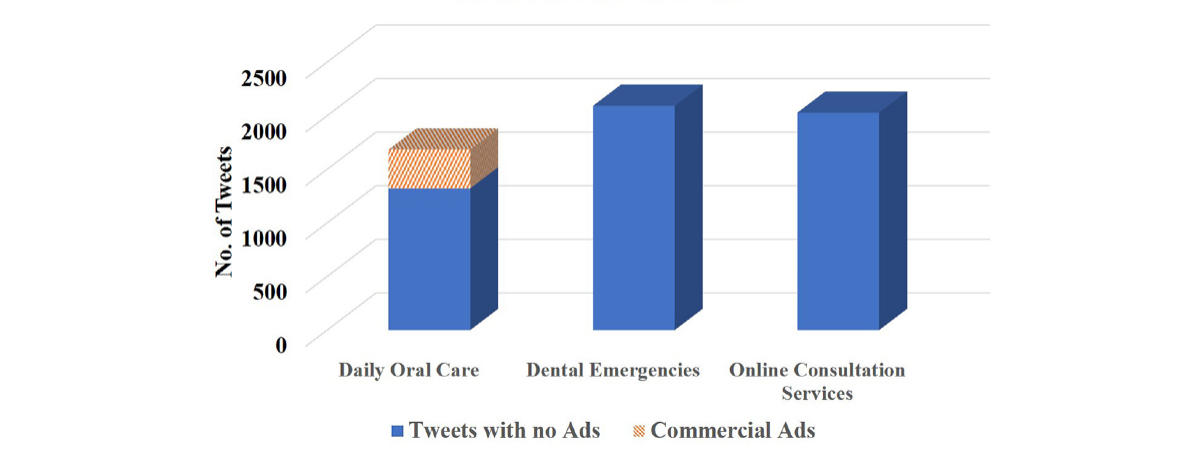
Thematic distributions of tweets with home oral care information.

Interestingly, numerous tweets with home oral care content were found on Weibo when the majority of dental services were not available for the public, much more than the tweets seeking oral health care as previously mentioned (4803 vs 2973 tweets). Information about daily oral care, how to deal with dental emergencies at home, and online consultation services shared similar proportions among these tweets (n=1684/4803, 35.06%; n=2092, 43.56%; and n=2029, 42.24%, respectively; Figure 7). Notably, 21.50% (n=362/1684) of daily oral care–related tweets presented commercial advertisements of oral hygiene products including toothbrushes, toothpastes, and dental floss.

As for the public responses to dental services and home oral care–related tweets from different types of bloggers, the number of likes, shares, and comments for tweets from governments, media, dental clinics and hospitals, dentists and dental nurses, online health platforms, and other nondental bloggers were counted and analyzed (only bloggers with >1000 followers were included; Table 3). Significant differences of public attention were found between various types of bloggers in dental service–related tweets (*χ*^2^_4_=113.883, 99.037, 49.544 for numbers of likes, shares, and comments, respectively, and *P*<.001 for all of them). The same findings were seen in tweets with home oral care content (*χ*^2^_5_=292.817, 186.265, 264.250, respectively, and *P*<.001 for numbers of likes, shares, and comments). The governments and media tweeted a large number of tweets related to dental services but fewer related to home oral care content (492 vs 98 tweets from governments, 805 vs 87 tweets from media, respectively) and received public responses with high average numbers of likes, shares, and comments in both categories. The government-generated tweets with dental service information received significantly more shares and comments but less likes than with home oral care content (dental services vs home oral care, *P*<.001 for numbers of likes, shares, and comments), while the media-tweeted information related to dental services garnered much less public attention compared to home oral care content (*P*<.001 for numbers of likes, shares, and comments). As for the tweets from dental clinics and hospitals, the public seemed to pay more attention to the tweets associated with home oral care information than dental services (*P*=.001 for numbers of likes and comments), though tweets in both categories were intensive. Dentists and dental nurses tweeted a mass of information related to oral health, and there were no significant differences between public responses for tweets with home oral care information and dental services (*P*=.37, *P*=.30, and *P*=.81 for numbers of likes, shares, and comments, respectively). Notably, nearly one-fifth of tweets with home oral care information were provided by online health platforms and garnered 4.05 likes, 1.28 shares, and 0.59 comments on average.

**Table 2:** Needs for dental treatment during COVID-19 epidemic.

Needs of dental treatment	Number of tweets (n=2793), n (%)^a^
Toothache or wisdom tooth problem	1132 (40.53)
Oral ulcer	264 (9.45)
Orthodontic problem	536 (19.19)
Implants or prostheses	31 (1.11)
Pediatric oral diseases	81 (2.90)
Oral cancer	41 (1.47)
Others or not specific	788 (28.21)

^a^The sum value of all parts is over 100% because some tweets mentioned more than one need of dental treatment.

**Table 3:** Comparison of public reactions to tweets with dental services and home oral care information from different types of bloggers.

Public reactions			Blogger categories										
			Governments		Media		Dental clinics/hospitals		Dentists/dental nurses		Online health platform		Others
**Count, n (%)**													
	DS^a^	492 (16.05)		805 (26.26)		563 (18.37)		363 (11.84)		0 (0)		842 (24.47)	
	HOC^b^	98 (2.95)		87 (2.62)		1049 (31.60)		874 (26.33)		660 (19.88)		551 (16.6)	
**Likes**													
	DS, mean (SD)	11.91 (68.00)		54.36 (1053.84)		1.66 (8.21)		2.47 (47.20)		N/A^c^		11.47 (78.58)	
	HOC, mean (SD)	14.35 (128.38)		168.10 (1110.52)		1.27 (11.09)		26.82 (377.06)		4.05 (64.39)		5.69 (38.82)	
	*Z* value	–6.858		–5.49		–3.259		–0.896		N/A		–14.75	
	*P* value	<.001		<.001		.001		.37		N/A		<.001	
**Shares**													
	DS, mean (SD)	2.75 (8.44)		4.63 (33.37)		0.88 (5.75)		0.49 (9.37)		N/A		2.37 (14.81)	
	HOC, mean (SD)	1.94 (8.27)		32.48 (182.30)		0.88 (6.75)		5.34 (42.89)		1.28 (17.34)		3.81 (49.31)	
	*Z* value	–5.943		–5.234		–0.565		–1.045		N/A		–10.06	
	*P* value	<.001		<.001		.57		.30		N/A		<.001	
**Comments**													
	DS, mean (SD)	3.97 (15.79)		7.43 (74.11)		0.70 (2.59)		0.42 (8.08)		N/A		4.15 (23.85)	
	HOC, mean (SD)	2.87 (23.22)		25.36 (154.88)		0.33 (1.56)		5.55 (51.73)		0.59 (8.85)		2.39 (12.42)	
	*Z* value	–5.925		–3.81		–3.279		–0.236		N/A		–12.68	
	*P* value	<.001		<.001		.001		.81		N/A		<.001	

^a^DS: dental services.

^b^HOC: home oral care.

^c^Not applicable.

## Discussion

### Principal Results

Since the COVID-19 outbreak exerted a negative impact on dental practices [12], the Chinese online community reacted rapidly and tweeted considerable information associated with COVID-19 and dentistry and oral health. This is the first study to analyze COVID-19–related oral health information that was presented on Chinese social media and evaluate the public interaction with this information. As shown in our study, COVID-19–related oral health content tweeted on Weibo may serve as a key indicator of the supply and demand of dental services under the epidemic in China.

### The Distribution of Tweets and the Epidemiology of COVID-19

It was interesting to note that the time distribution of the Weibo data was approximately consistent with the trend of both daily new cases and deaths. Our finding is similar to a previous study on H7N9–related tweets on Weibo, which identified a positive correlation between the number of daily tweets and the cumulative case fatality rate of H7N9 [26]. Accordingly, the time distribution of social media may help predict the trend of new cases and deaths during the epidemic, thus, serving as a cost-effective and useful tool for epidemiology study.

Additionally, the peaks of tweets were influenced by some milestone events of COVID-19, including the official confirmation of human-to-human transmission of COVID-19, lockdown of Wuhan, and the death of Dr Wenliang Li. The possible explanation for this phenomenon is that social media engages more public attention during outbreaks of EIDs, especially when important news about the epidemic is released [27]. Similarly, during the H1N1 pandemic in 2009 and the outbreaks of MERS-CoV in 2012 and H7N9 in 2013, the time distribution of related tweets on social media were impacted by milestone events, such as the official announcement of the first case diagnosed and the level of the epidemic [17,28].

Although our results show that the geographical distribution of the tweets was roughly consistent with the distribution of total COVID-19 cases, Hubei Province was an exception. As the first epicenter of a newly identified infectious disease in the world, people may have been overwhelmed, and the concerns for oral health problems and demands for dental services were unavoidably suppressed. This phenomenon should attract more attention from public health policy makers.

Social media should not only provide true and useful information for the public, but also possess self-correction function for misinformation [29]. As illustrated in our study, during the early stage of the COVID-19 outbreak, some misleading information for prevention of COVID-19 was diffused among the online community, which attributed to a natural fear for the unknown and unexpectable disaster. This phenomenon is quite common during outbreaks of emerging diseases. When yellow fever re-emerged during 2015-2017, nearly two-thirds of tweets associated with yellow fever contained misinformation including some improper treatments [30]. After the first Zika infection case confirmed in the United States, grassroots users on Twitter amplified social concerns and even tweeted conspiracy theories [31]. A systemic review about social media and outbreaks of EIDs showed that 20%-30% of the EID–related YouTube videos contained inaccurate or misleading information [27]. In our study, the refutations of misinformation were tweeted by the official platform “Weibo Refutes Rumours” and immediately exchanged by other users with a larger scale on Weibo. This may contribute to calming the public panic with scientific knowledge.

### Dental Services and Dental Care Needs During the COVID-19 Outbreak

Social media can amplify the spread of contents compared to traditional mass media [32]. In this study, the most widely diffused information was about aerosol as a transmission route of COVID-19 and its effect on dental practices, which was first tweeted on January 25, 2020, on Weibo (earlier than scientific publications). The study that first confirmed SARS-CoV-2 existed in saliva was published on February 12, 2020 [4], and the study that first announced the possibility of COVID-19 transmission by aerosol-generating dental procedures was published on February 20, 2020 [9]. The updates and spread of information on social media are updated to the minute, and the communication on social media is in real time, much faster than traditional media and online scientific publications. Moreover, increasing numbers of bloggers in public health professions are active on social media and tweet scientific knowledge on epidemics of infectious diseases, which plays a crucial role in shaping public awareness and response to an emerging disaster [33].

The analysis of the COVID-19–related oral health contents on Weibo provided an overview of the supply and demand of dental services during the COVID-19 outbreak. The public concern about the risks of spreading coronavirus by aerosols was disseminated earlier than the official notices of stopping part of or all dental services. With insufficient dental services, the public needs for oral care could not be satisfied, and an increased number of users complained of oral diseases or sought for consultation on Weibo. Our study showed dynamic changes of information related to supply and demand of dental services during this period, indicating that social media can serve as a useful tool for the monitoring of medical and health demands during an unprecedented time [34].

Social media plays an increasingly important role in health policy making [20,21,35]. As shown in our review, the urge of stopping dental services to avoid potential risks of coronavirus transmission between patients and dental care providers was tweeted by dental professionals and retweeted by other bloggers at the end of January, which may contribute to the public health policy about suspension of dental practices from the governmental agencies at the beginning of February. This important function of social media benefits from the multidirectional conduit of social networks, leading to more efficient and wide diffusion of information on social media than on traditional media [36] and more frequent interactions between individuals and public health organizations and policy makers [37]. Regarding social media as a rapidly maturing channel of communication, the policy makers can obtain evidence to help make health policy decisions, disseminate the policies on social media, and monitor the public reactions to the policies [38].

In response to perceived unmet dental care needs from the online community, home oral care information was highly tweeted on Weibo to satisfy the public needs for both daily oral care and dental emergencies. Remote dental consultations were also achieved through Weibo, not only providing diagnosis and suggestions for patients with oral health problems but also avoiding risks of coronavirus transmission. This correlation between dental service disruption and an increased use of social media as a means of communication is not uncommon during a disaster or emergency [39]. Previous studies have also recommended social media for dental public health surveillance due to its potential in monitoring episodes of dental pain [40,41]. The active exchange of information related to oral care and the interaction between patients and dental professionals were found from our Weibo data, presenting a promising communication mode between health care providers and patients as a supplement for the traditional doctor-patient relationship.

### The Variation of Public Reactions to Tweets From Different Bloggers

In our study, the online community reacted variously to the tweets from different bloggers. The tweets posted by governments and media attracted more responses from the public due to their authority [42]. The government and media should take the responsibility to tweet true and real time information. Surprisingly, the influence of dental clinics and hospitals, and dentists and dental nurses was not strong enough despite tweeting high-quality information of oral health. The important role of the emerging online health platforms was identified in this study. The online health platforms with more followers than individual dental professionals can transmit health-related information more efficiently and benefit more people [43]. From the perspective of social equality, the services from online health platforms can also cover those hard-to-reach populations, making it an equitable access to health care for the public. Therefore, our results suggest that the online health platform has potential to be a promising solution for cost-efficient health care information and medical consultations.

### Limitations

Several limitations of our study should be acknowledged. First, there was inevitable bias of information in the data collection process. For example, Weibo is more popular among young people rather than the aged and is more accessible for economically developed regions. Therefore, the impact of age and regional distribution of users may need to be considered when interpreting the results. As a real time social media, some tweets were deleted by bloggers and some bloggers’ accounts were suspended by Sina Weibo, which led to direct information loss. In the geographic analysis of tweets, around 90% did not provide location information, thus affecting the overall objectivity and accuracy of results to a certain extent. Second, our study did not provide any data on the characteristics of Weibo users who viewed and shared these tweets, and the complete diffusion route of tweets were not extracted and analyzed. Therefore, the audience of the information and the diffusion scale of tweets could not be accurately evaluated. Third, the information provided by online consultation services was not available, and thus, the quality of online consultations and the effects on the patients remained unknown.

### Conclusions

To the best of our knowledge, this is the first study to comprehensively overview and analyze social media data on the dental services and oral health information during the COVID-19 epidemic in China. Based on our results, it is evident that social media users reacted immediately to the emerging SARS-CoV-2 threat to dental practices. Social media not only contributed to public health surveillance and policy making but also served as a bridge between oral health information providers and the patients. The findings illustrate the relationship between social media information with the supply and demand of dental services during the outbreak of the COVID-19 epidemic in China. In addition, the study provides insights for government organizations, media, and dental professionals to efficiently affect and shape public awareness, and disseminate dental public health information through social media.

## Appendix

Multimedia Appendix 1 Thematic distribution of tweets related to oral health/dentistry during the coronavirus disease epidemic, December 31, 2019, to March 16, 2020.

## Abbreviations

CCDC: Chinese Center for Disease Control and Prevention
COVID-19: coronavirus disease
EID: emerging infectious disease
MERS-CoV: Middle East respiratory syndrome–related coronavirus
SARS-CoV-2: severe acute respiratory syndrome coronavirus 2
