# Supplementary material for: Nature and Diffusion of COVID-19–related Oral Health Information on Chinese Social Media: Analysis of Tweets on Weibo
Source: J Med Internet Res. 2020 Jun 15;22(6):e19981. doi: 10.2196/19981 (PMC7296975; doi:10.2196/19981)
Supplement: Multimedia Appendix 1 [file jmir_v22i6e19981_app1.docx]

Supplementary Table 1. Thematic distribution of tweets related to oral health/dentistry during COVID-19 epidemic, 31st December 2019 – 16th March 2020.

| **Thematic categories** | | **Definition** | **Example** |
| --- | --- | --- | --- |
| **COVID-19 related information** | |  |  |
|  | Epidemiology | The determinants and distribution of COVID-19 in defined populations, e.g., infection sources, transmission routes, susceptible population, etc. | [There are 3 specific transmission routes of COVID-19] 1. Direct transmission: cough, sneeze, droplets and even exhalations from patients will lead to infection after inhalation. 2. Aerosol transmission: droplets mix with the air and form aerosols, leading to infection after inhalation. 3. contact transmission: hands contact droplets on the surface of objects, and then contact with oral, nasal, and eye mucous membranes, resulting in infection. |
|  | Pathology | The causes and effects of COVID-19, e.g., infection mechanisms, specific tissue/cell alterations after infected, etc. | The receptor of novel coronavirus is angiotensin-converting enzyme 2 (ACE2), the same host receptor for SARS-CoV, which means that the infection starts when the virus contacting the cell with the enzyme and binding to the receptor… We have quite a few of these cells exposed to the air -- the mucous membrane. We have a lot of mucosal cells in our lips, eyelids, nose and oral cavity, and when the virus contacts your oral mucosa and binds to the receptor, the infection begins. |
|  | Symptoms | Departures from normal functions or feelings in COVID-19 patients. | The early symptoms of COVID-19 patients are a bitter taste in the mouth and loss of appetite in the oral cavity, which are indirect evidence of the coronavirus infecting the mouth… |
|  | Prevention | Measures to protect individuals from COVID-19, including wearing masks, washing hands, social distancing, and even some incorrect methods and refutations for the misinformation, etc. | 1. Masks can prevent virus infection by preventing droplets from the oral and nasal cavity. Correctly choosing and wearing a surgical mask or medical protective mask, not only can protect ourselves from infection, but also protect others when we have respiratory symptoms. # prevention of COVID-19# 2. Frequent hand washing and disinfecting, and avoiding touching the mucous membrane (such as eye, nasal and oral mucosa, etc.) with contaminated skin, can effectively prevent the spread of the virus. 3. During the epidemic, please, keep your contact with outsiders to a minimum... 4. [Academician Zhong Nanshan suggested gargling with salt water to prevent viruses? It’s a rumor!] Zhong’ team refuted the rumor, “Gargling with salt water is good for cleaning the mouth and throat and is helpful for laryngitis, but cannot clean the respiratory tract where the coronavirus invades human. Secondly, to date, there are no evidence suggesting that salt water can kill the novel coronavirus.” |
|  | Diagnosis | The process/methods of determining SARS-CoV-2 infection, e.g., oral or throat swab for virus detection | The application of antibody tests to track the source of infection in new cases is a major innovation in the fight against the epidemic." Currently, novel coronavirus detection uses polymerase chain reaction (PCR) to investigate genetic material of the virus from saliva, nasal, oral, or anal swabs," *Science* reported. |
| **Oral health related information** | |  |  |
|  | Dental services during COVID-19 epidemic | - Risks of COVID-19 spread during dental procedures | The diagnosis and treatment of stomatology department is special. Dental handpieces produce a lot of aerosol, and if a COVID-19 patient in incubation period receives dental treatment, it is easy to lead to explosive spread of the epidemic. So please delay the treatment if you are not with maxillofacial infection, trauma, or other dental emergencies. |
|  |  | - Notices of stopping all or part of dental services | [Notice: stop dental services of dental clinics in the province] On January 30, leading group office for COVID-19 epidemic prevention of Jilin province issued the notice “standard of dental services during COVID-19 epidemic”. The notice requires all the dental clinics to stop providing dental services except the necessary dental emergency treatment. |
|  |  | - Protective measures to cut off/ decrease COVID-19 transmission in dental practices | All Sanye dental clinics have started emergency protection against COVID-19 epidemic. 1) All clinics are equipped with quick-drying hand sanitizer, and all outpatients should wash hands. 2) Infrared thermometers are used to measure the body temperature of each patient, including adults and children. 3) Public areas and toys are disinfected. 4) Surgical masks are available in all clinics and free of charge for both visitors and residents in neighbor. 5) A pre-examination desk is set up at the gate. All the visitors entering the clinics should provide personal information and temperature first. |
|  |  | - Notices of restoring dental services. | In order to prevent the epidemic scientifically and meet the demand for dental emergency treatment of the public, the west China stomatological hospital of Sichuan University will restore its general outpatient service form March 9. The hospital will strictly manage the outpatient entrances and exit. |
|  | Needs of dental treatment | The public needs of dental services during the epidemic, such as toothache, orthodontic problem, etc. | 1. Toothache woke up me at midnight. When will the antibiotics be delivered to me? I will go to the stomatological hospital as soon as the epidemic ends. 2. One of my brackets had fallen off and wire broken during the spring festival. I am worried about the recrudescence of my teeth as dental clinics are closed during the epidemic. |
|  | Home oral care | - Daily oral care tips, including some commercial ads for oral hygiene products. | How to keep oral health during the epidemic? 1. Brush your teeth in the morning and evening, and gargle with water after meals. 2. Use dental floss, or an interdental brush to help clean the teeth. 3. Eat sugar in moderation. Reduce times for eating sugar every day and drink less carbonated drinks. 4. Protect your teeth and use teeth correctly. 5. Eat a balanced and nutritious diet, and avoid hard and hot food. 6. Take good care of the elderly and children to prevent falls and injuries. |
|  |  | - Tips on how to deal with dental emergencies at home. | During the epidemic period, how to deal with acute pericoronitis when the dental services are not available? 1. Light diet. Gargle and use floss after meals. 2. Gargle with salt water or mouthwash several times a day. 3. If necessary, take amoxicillin (or cephalosporin antibiotics) + tinidazole (or metronidazole) to relieve the symptoms. |
|  |  | - Online consultation services provided by dentists, dental clinics/hospitals, online health platforms, etc. | In order to ensure the oral health of all patients during the epidemic, Yafei dental clinic has invited professors from West China Medical University to Yafei online clinic. Welcome to consult for oral problem at 3:00-6:00 P.M., 9^th^ Feb. |
| **Others** | |  |  |
|  | Other COVID-19-related oral health information but cannot be sorted into the themes mentioned above, e.g., shortage of protective supplies for dentists, promotional spots of dental clinics, etc. | | 1. Recently, the protective supplies of all dental medical institutions in Wuhan are very limited. The Chinese stomatological association proposes that medical colleges, medical institutions and relevant enterprises offer a helping hand and donate some necessary supplies to make contributions to the prevention of the epidemic. Let's pull through together! 2. During the outbreak of COVID-19, Mei’ao Dental Clinic is carrying out a promotion that￥499 buying ￥1000 stored-value card. You can enjoy half-price discount in our clinic for all kinds of services! |
